# Assessing cortical plasticity after spinal cord injury by using resting-state functional magnetic resonance imaging in awake adult mice

**DOI:** 10.1038/s41598-018-32766-8

**Published:** 2018-09-26

**Authors:** Kohei Matsubayashi, Narihito Nagoshi, Yuji Komaki, Kota Kojima, Munehisa Shinozaki, Osahiko Tsuji, Akio Iwanami, Ryosuke Ishihara, Norio Takata, Morio Matsumoto, Masaru Mimura, Hideyuki Okano, Masaya Nakamura

**Affiliations:** 1 Department of Orthopaedic Surgery, Keio University School of Medicine, Tokyo, Japan; 2 Department of Physiology, Keio University School of Medicine, Tokyo, Japan; 3 Central Institute for Experimental Animals, Kawasaki, Japan; 4 Department of Neuropsychiatry, Keio University School of Medicine, Tokyo, Japan; 5 Laboratory for Marmoset Neural Architecture, RIKEN Center for Brain Science, Wako-shi, Saitama 351-0198 Japan

## Abstract

Neural connectivity has recently been shown to be altered after spinal cord injury (SCI) not only in the spinal cord but also in the brain. However, to date, no studies have analyzed the functional alterations after SCI in various areas of the cerebral cortex over time. To examine the plasticity of the neural connectivity in the brain after SCI, we performed resting-state functional magnetic resonance imaging (rs-fMRI) in awake adult mice pre- and post-SCI. After a complete thoracic SCI, the functional connectivity between the primary motor (MOp) and primary sensory (SSp) areas was significantly decreased during the chronic phase. In contrast, the connectivity between the MOp and motivation area was increased. Thus, impairments in sensory and motor connections after SCI led to a time-dependent compensatory upregulation of “motor functional motivation”. Moreover, the functional connectivity between the SSp and pain-related areas, such as the caudoputamen (CP) and the anterior cingulate area (ACA), was strengthened during the chronic phase, thus suggesting that rs-fMRI can indicate the presence of neuropathic pain after SCI. Therefore, rs-fMRI is a useful tool for revealing the pathological changes that occur in the brain after SCI.

## Introduction

With the development of magnetic resonance imaging (MRI) techniques, the functional and structural reorganization of the brain after spinal cord injury (SCI) have been clarified. For example, a previous study using MRI voxel-based morphometry (VBM) has revealed a decrease in the volume of the primary motor (MOp) and sensory (SSp) areas in patients with SCI compared with healthy subjects^1,2^. In another study, a functional MRI (fMRI) analysis performed using a cervical SCI rat model has revealed signal changes in the region of the cortex that controls forelimb movement^3^.

Resting-state functional MRI (rs-fMRI) is superior to other imaging methodologies, owing to its ability to record spontaneous fluctuations in the blood-oxygen-level dependent (BOLD) signal at rest. The functional connectivity among different brain regions can be evaluated by calibrating the degree of the correlation among these regional fluctuations. Previous clinical studies using this technique have revealed significant changes in the visual cortex network in patients with chronic SCI^4^ and a significant increase in the functional connectivity between the primary somatosensory area and supplementary motor area in patients with acute SCI compared with healthy subjects^5^. However, these studies have some limitations because (1) the authors did not record the changes in the connectivity in the same individual over time and (2) the SCI populations were heterogenous in terms of the patient demographics and the level and severity of the SCI. Because of these limitations, comprehensive analysis of rs-fMRI data is difficult. To eliminate these inconsistencies, we established a mouse model with a complete thoracic SCI and performed rs-fMRI in the awake state over time from the acute stage to the chronic stage.

## Results

### Changes in the functional connectivity in the entire brain of awake mice after SCI

**Figure 1:**
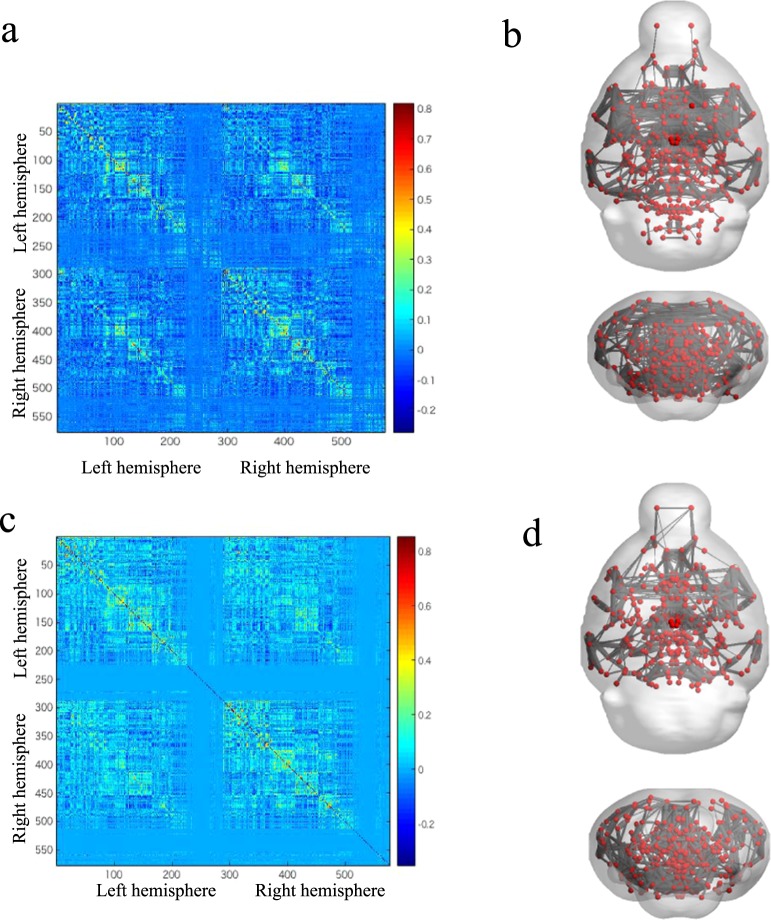
Visualization of the neuronal functional connectivity in an awake mouse. (**a**) The correlation matrix of awake mice with intact spinal cords expressed as the correlations among 576 areas. (**b**) The brain networks were visualized on the basis of the correlation matrix. The nodes represent the region of interest (ROI) positions, and the edges represent the correlations among the nodes. (**c**) The correlation matrix of awake mice 14 weeks after the SCI. (**d**) The brain networks were visualized on the basis of the correlation matrix of mice 14 weeks after the SCI.

Although several studies have demonstrated the functional connectivity in mouse brains by using fMRI, we sought to evaluate whether similar results could be observed through this imaging technique during the awake state in mice with SCI. We constructed a correlation matrix that indicated the functional connectivity between specific regions (Fig. 1a). Furthermore, we successfully visualized the connectivities on the basis of the correlation matrix (Fig. 1b). Therefore, using a similar method, we constructed a correlation matrix of the post-SCI brain (Fig. 1c,d). Similar matrix were drawn for the sham group (Supplement Fig. 1a,b)

**Figure 2:**
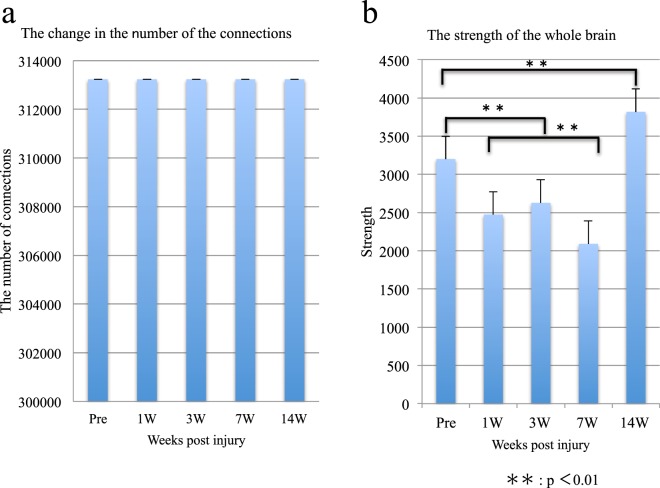
Alterations in the connectivity in the entire brain. (**a**) The change in the number of connectivities in the entire brain. (**b**) The change in the strength of the connectivities in the entire brain.

Then, we analyzed the changes in the functional connectivity in the entire brain after SCI. From the pre-SCI stage to 14 weeks post-SCI, the total number of connections in the brain did not change (Fig. 2a). However, we observed a decrease in the connectivity strength 7 weeks after the SCI, as compared with the pre-SCI values. Subsequently, by week 14, the connectivity had significantly increased (Fig. 2b). Furthermore, when Network based statistics was performed using graph theory, characteristic path length tended to increase at 14 weeks post -SCI compared with before the SCI, but clustering coefficient tended to not change before and post the SCI. However, there was no statistically significant difference (Supplement Fig. 2a,b).

On the other hand, there was no significant change of the strength in the sham group before and after the sham operation (Supplement Fig. 3a,b).

### Reorganization of the functional connectivity between the MOp and SSp/ motivation areas

**Figure 3:**
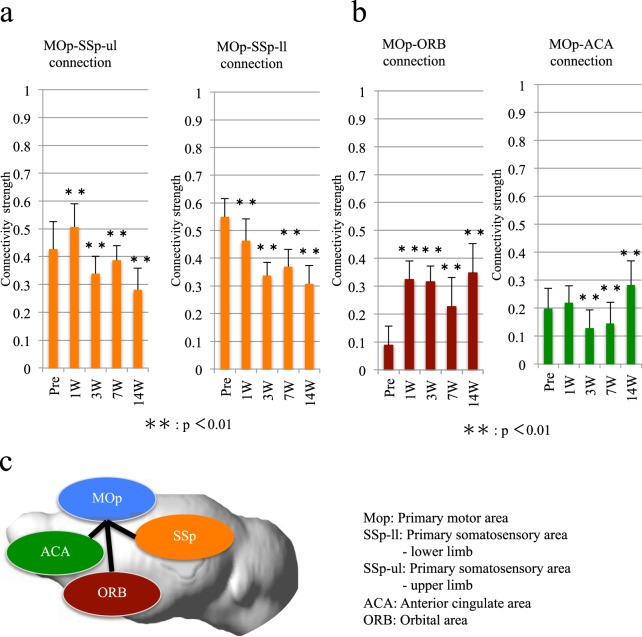
The connectivities between the MOp and the SSp, ORB and ACA. (**a**) The change in the strength of the connectivity between the MOp and the SSp. (**b**) The change in the strength of the connectivity between the MOP and the CP/ACA. (**c**) A schematic of the connections among the MOp and the SSp, ORB and ACA.

Signal changes have been shown to occur in the MOp and SSp after SCI, owing to motor and sensory neurological deficits^6^. However, in that study, the authors did not investigate the connectivity. Therefore, we investigated the local changes in the functional connectivity in these areas in awake mice with SCI. One week after the injury, the strength of the functional connectivity between the MOp and the upper limb SSp areas was significantly increased (Fig. 3a). However, the strength of this functional connectivity was significantly decreased at week 3 and remained lower than the pre-SCI value during the entire 14-week follow-up period. In contrast, the strength of the connectivity in the lower limb SSp region was significantly decreased one week after the SCI and continued to decrease over time (Fig. 3a).

Then, we evaluated the connectivity between the MOp area and the orbital area (ORB) and the anterior cingulate area (ACA), both of which are related to motivation. We focused on these areas because a previous electrophysiological study has demonstrated a strengthened connection between the MOp and other motivation areas in a non-human primate SCI model^7^. Consistently with the results of the previous study, in our experiment, the strength of the functional connectivity between the MOp and the ORB was significantly increased one week after the injury and was maintained at this higher strength level until week 14. However, the strength of the functional connectivity between the MOp and the ACA was significantly decreased at weeks 3 and 7 after the SCI, and this decrease was followed by a significant increase at week 14 to a level that was well above the initial preinjury level (Fig. 3b).

Moreover, there was no significant change of the strength in the sham group before and after the sham operation (Supplement Fig. 4a,b).

### Plasticity of the functional connectivity between the SSp and pain-related areas

**Figure 4:**
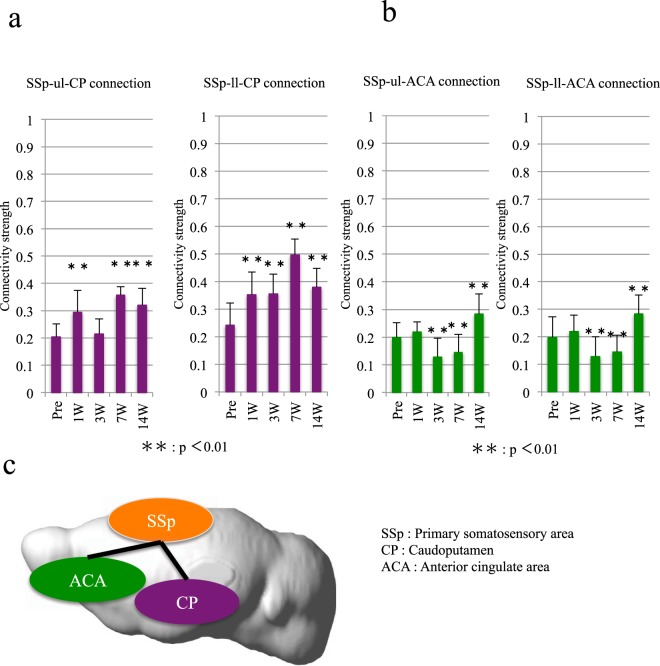
The connectivity between the SSp and the CP/ACA. (**a**) The change in the strength of the connectivity between the SSp and CP. (**b**) The change in the strength of the connectivity between the SSp and ACA. (**c**) A schematic of the connections between the SSp and CP/ACA.

Subjects with sustained pain have been shown by rs-fMRI to exhibit stronger connectivities between the SSp and other brain regions, such as the caudoputamen (CP) and the ACA^8^. Therefore, these areas are considered to be related to pain. Using rs-fMRI to examine these areas in awake mice, we found that the connection between the SSp and the CP was mostly upregulated over time and reached levels higher than those at the preinjury time point (Fig. 4a,c). The connection between the ACA and the upper and lower limb SSps was significantly decreased at weeks 3 and 7 and then significantly increased at week 14 (Fig. 4b,c).

There was no significant change of the strength in the sham group before and after the sham operation (Supplement Fig. 5a,b).

## Discussion

Using rs-fMRI on awake SCI mice, we successfully revealed the plasticity of the functional connectivity in the brain over time. Although the number of connections in the whole brain did not change, the strength of individual connectivities varied over time. The reorganization of the functional connectivity is speculated to be attributable to the changes in the connectivity strength. In particular, the strength of the connectivity between the MOp and SSp was decreased, but the connectivity strength among the motivation areas was increased during the chronic phase. These results may reflect a compensatory enhancement of “motor functional motivation” due to the loss of motor and sensory connections after SCI. Moreover, the strength of the connectivity between the SSp and the pain-related areas was significantly increased compared with that during the preinjury condition, thus probably indicating the occurrence of neuropathic pain during the chronic phase. Therefore, rs-fMRI is a potentially useful tool for the detection of time-dependent alterations in brain functions.

Previous studies involving humans and rats have produced correlation matrices of the entire brain by using rs-fMRI^9,10^. In this study, we also successfully constructed a correlation matrix for the mouse brain, thus enabling the analysis of the functional connectivity in the brain. This animal model has several advantages including that: (1) genetic modification tools are easily available and (2) various disorder/trauma models have been established to investigate therapeutic efficacies, such as cell transplantation and drug administration^11^. It is of great importance to conduct further analyses of functional connections in combination with these models.

The strength of the functional connectivity between the MOp and SSp was significantly decreased 14 weeks after the injury (Fig. 3a). This result was similar to those obtained in previous studies that performed rs-fMRI on human patients with chronic SCI^12^. The decrease in the connectivity in both mice and humans was explained by the decrease in the sensory input from the trunk and extremities and the loss of motor function. Therefore, rs-fMRI may be useful for assessing the effects of treatments, such as rehabilitation techniques, that promote sensory and motor coordination.

We evaluated the strength of the functional connectivities among the MOp and ORB and ACA and observed a significant increase in strength 14 weeks after the injury (Fig. 3b). Because neurological improvements were not apparent in our transected SCI model, this change may have resulted from efforts to move the hind limbs. Interesting findings were also observed in regard to the relationship between the SSp and the CP/ACA. For example, the connectivity between the SSp of the lower limbs and the pain-related areas during the chronic phase was increased (Fig. 4a), a result suggesting pain below the level of the injury. Moreover, although we established an SCI model at the thoracic level, we observed stronger pain-related signals in the upper limbs, which may have indicated post-SCI pain above the injury level (Fig. 4a,b). Previous studies have evaluated the manifestation of pain above the level of injury in rodent SCI models, but those studies have focused on only the mechanism of the SCI^13,14^. Our findings suggested that it is possible to detect this pain in the cerebral cortex. Hence, further investigations examining the association of this pain-related connection by using rs-fMRI with actual sensory abnormalities, such as neuropathic pain, are crucial.

Recently, graph theory has been applied to understand the functional connectivity in MRI, especially in the field of Alzheimer’s disease^15^ and Parkinson’s disease^16^. In contrast, there were few studies which used that theory for SCI. A recent representative study compared the network based statistics using graph theory in SCI patients to healthy controls, and demonstrated increase of characteristic path length and reduction of clustering coefficient at longer-term follow-up 58.3 ± 52.0 weeks after injury^17^. Our results using mouse SCI model presented similar trend with increased path length, whereas clustering coefficient was comparable before injury and at week 14 as final observational time point. The discrepancy between our study and theirs could be explained due to different animal species, sample size, and observational durations. Further study should be necessarily performed using larger sample size of mouse with long follow-up duration to evaluate whether the data from human SCI is able to be replicated.

There are several limitations to this study that should be noted. First, the relationships among behavior, recovery and changes in functional connectivity in the brain could not be assessed, because we used a complete transection model. Therefore, future studies should focus on motor function and analyze incomplete contusion injury models that exhibit some spontaneous recovery. Furthermore, although the results of this rs-fMRI study indicated the existence of functional connectivity, comparative studies that include histological analyses remain to be performed. Although it has been pointed out in past papers^18^, it is essential to focus on the association between connectivity and histology. Further studies to evaluate the relationship between fMRI and DTT, fMRI and tracer imaging will be needed in the future. In conclusion, we successfully visualized the functional connections in the entire brain in mice and quantitatively evaluated the strength of the connectivities among various areas by using rs-fMRI. This method may serve as a valuable tool for analyzing pathological changes in the brain after SCI.

## Methods

### Animals

Nine female C57BL/6 J mice (8 weeks old, 18–20 g weight, CLEA Japan Inc.) were used in this study. All animal experimental procedures were performed in accordance with the Laboratory Animal Welfare Act and the Guide for the Care and Use of Laboratory Animals (National Institutes of Health, Bethesda, MD, USA). All experiments were approved by the Animal Study Committee of the Central Institute for Experimental Animals (approval number: 14078).

### Surgical procedure for attaching the head-bar

The mice were deeply anesthetized with a combination of ketamine (100 mg/kg) and xylazine (10 mg/kg) and then fixed to a stereotaxic apparatus. An incision was made, and the skull surface was exposed. The surface was covered with a thin layer of dental cement with a brush-up technique (Super-Bond C&B, Sun Medical, Shiga, Japan). A custom-made acrylic head-bar (3 × 3 × 27 mm^3^) was mounted along the sagittal suture of the exposed skull using dental cement. The mice were fed a high-energy diet.

### Surgical SCI procedure

The spinal cords of the mice were transected after the head-bar was attached. The mice were anesthetized with ketamine (100 mg/kg) and xylazine (10 mg/kg). After the laminectomy at the level of the Th9/10 vertebra, the dorsal surface of the dura mater was exposed, and an incision was made. The spinal cord was then transected with a single incision using a surgical blade. The severed ends of the cord were carefully inspected under a surgical microscope to ensure complete transection. For the operation of sham group, we performed only laminectomy at the level of Th9/10. Functional changes after SCI were evaluated using Basso Mouse Scale^19^ (Supplement Fig. 6).

### rs-fMRI in awake mice

rs-fMRI was performed on awake mice as previously described^20^. To perform rs-fMRI on awake mice, the mice were habituated. In this experiment, rs-fMRI was performed before the injury and 1, 3, 7 and 14 weeks after the injury, and the mice were habituated each time for 1 week prior to undergoing rs-fMRI.

rs-fMRI was performed using a 7.0 tesla MRI system equipped with actively shielded gradients at a maximum strength of 700 mT/m (Biospec; 70/16 Bruker BioSpin, Ettlingen, Germany) with a cryogenic quadrature radio frequency (RF) surface probe (CryoProbe; Bruker BioSpin AG, Fällanden, Switzerland) to improve the sensitivity^21–24^. The animal respiration was monitored while the rs-fMRI was performed. The scan protocols used for the rs-fMRI have been previously described^25^. To construct a reference for the brain anatomy, high-resolution T_2_-weighted images (T_2_WIs) of the whole brain were acquired using a Rapid Acquisition with Relaxation Enhancement (RARE) method with the following parameters: effective time to echo (TE), 48 ms; time to repetition (TR), 6100 ms; RARE factor, 8; number of averages, 4; spatial resolution, 75 × 75 × 300 μm^3^; and number of slices, 52. The BOLD fMRI signal was acquired using a gradient echo-echo planar imaging method with the following parameters: TE, 20 ms; TR, 1000 ms; flip angle, 55°; number of averages, 1; spatial resolution, 200 × 200 × 500 μm^3^; and number of slices, 16. The scan was repeated 600 times in 10 minutes.

### Data analysis

We took rs-fMRI for nine mice each twice and compared the mean. The rs-fMRI data analysis was performed using SPM12 (Wellcome Trust Centre for Neuroimaging, UCL Institute of Neurology, London, UK) and tailored software for MATLAB, which allowed for adjustments for the slice acquisition timing, motion correction, and co-registration of the different mouse brains to the stereotaxic MRI brain template for intact C57BL/6 J mice. The normalized functional images were smoothed with a 0.6-mm full-width at half-maximum (FWHM) filter. The functional connectivity analysis was performed using CONN^26^. A temporal bandpass filter was applied in the 0.009 Hz to 0.1 Hz range^27^. These data analysis methods were based on a previous article^25^. The functional connectivity among areas were analyzed using the regions defined in the Allen Brain Atlas^28,29^. Five hundred and seventy-six regions were defined. The brain networks were visualized using BrainNetViewer^30^, http://www.nitrc.org/projects/bnv (accessed 2017-03-03).

To analyze the functional connectivity of the entire brain, the sum of the functional connectivity within the entire brain was obtained by adding the individual functional connectivity. The total strength of the functional connectivities in the whole brain was obtained by adding all the strengths of the functional connectivity in different parts of the brain. The functional connectivity among individual regions on the same side of the brain were calculated. To compare the changes in the respective strengths after the SCI, statistical analysis was performed with MATLAB and SPSS version 23 (IBM, Armonk, New York, USA). We first check the normality and do Dunnett’s test to compare the significant differences. A graph theory analysis based on an undirected, weighted connection matrix was performed with the Brain Connectivity toolbox^31^.

## Electronic supplementary material

Supplementary Material
