# Supplementary material for: Assessing cortical plasticity after spinal cord injury by using resting-state functional magnetic resonance imaging in awake adult mice
Source: Sci Rep. 2018 Sep 26;8:14406. doi: 10.1038/s41598-018-32766-8 (PMC6158265; doi:10.1038/s41598-018-32766-8)
Supplement: Supplementary file 1 — Supplementary Material [file 41598_2018_32766_MOESM1_ESM.pdf]

Assessing cortical plasticity after spinal cord injury by using resting-state functional magnetic resonance imaging in awake adult mice

Kohei Matsubayashi[1,2], Narihito Nagoshi[1], Yuji Komaki[2,3], Kota Kojima[1], Munehisa Shinozaki[2], Osahiko Tsuji[1], Akio Iwanami[1], Ryosuke Ishihara[4], Norio Takata[4], Morio Matsumoto[1], Masaru Mimura[4], Hideyuki Okano[2,5,*], Masaya Nakamura[1,*]

[1]Department of Orthopaedic Surgery, Keio University School of Medicine, Tokyo, Japan

[2]Department of Physiology, Keio University School of Medicine, Tokyo, Japan

[3]Central Institute for Experimental Animals, Kawasaki, Japan

[4]Department of Neuropsychiatry, Keio University School of Medicine, Tokyo, Japan

[5]Laboratory for Marmoset Neural Architecture, RIKEN Brain Science Institute, Wako-shi, Saitama, 351-0198, Japan

***Corresponding authors:**

Masaya Nakamura, Department of Orthopaedic Surgery, Keio University School of Medicine, 35 Shinanomachi, Shinjuku, Tokyo, 160-8582, Japan.

Tel.: ±81-3-5363-3812**,** Fax: ±81-3-3353-6597**,** E-mail: masa@keio.jp

Hideyuki Okano, Department of Physiology, Keio University School of Medicine, 35 Shinanomachi, Shinjuku, Tokyo, 160-8582, Japan.

Tel.: ±81-3-5363-3747, Fax: ±81-3-3357-5445, E-mail: hidokano@a2.keio.jp

Supplement Figure 1

a

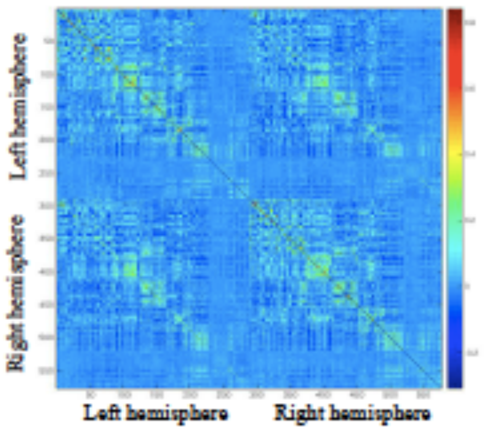

b

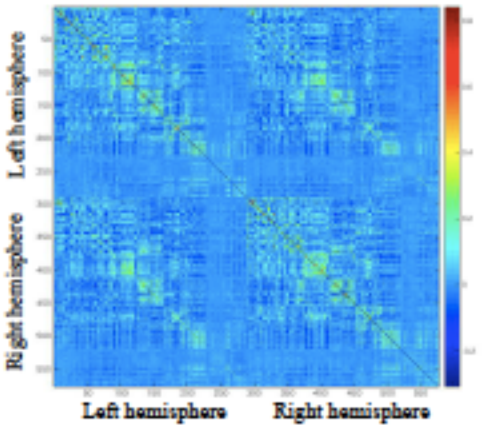

Supplement Figure 2

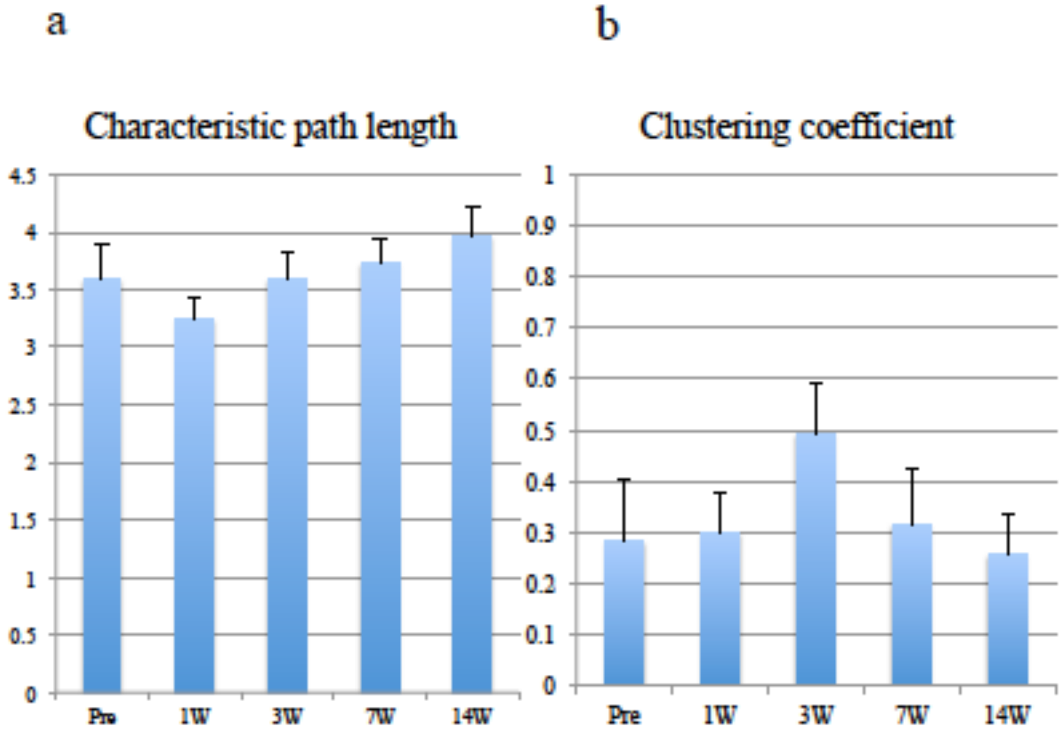

Supplement Figure 3

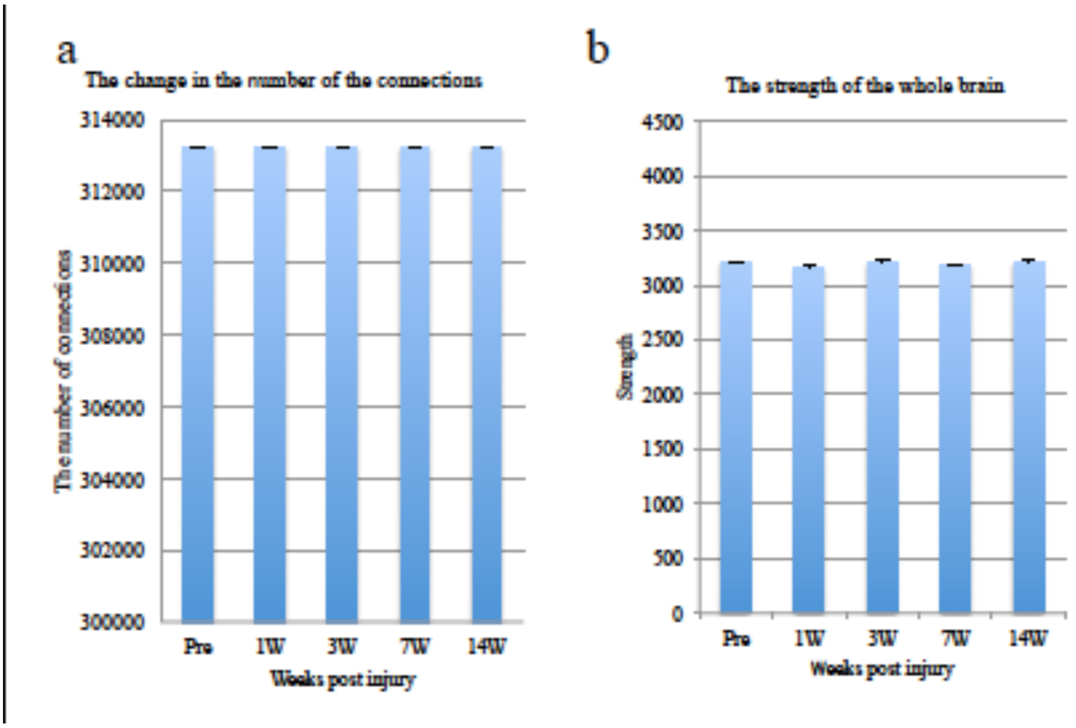

Supplement Figure 4

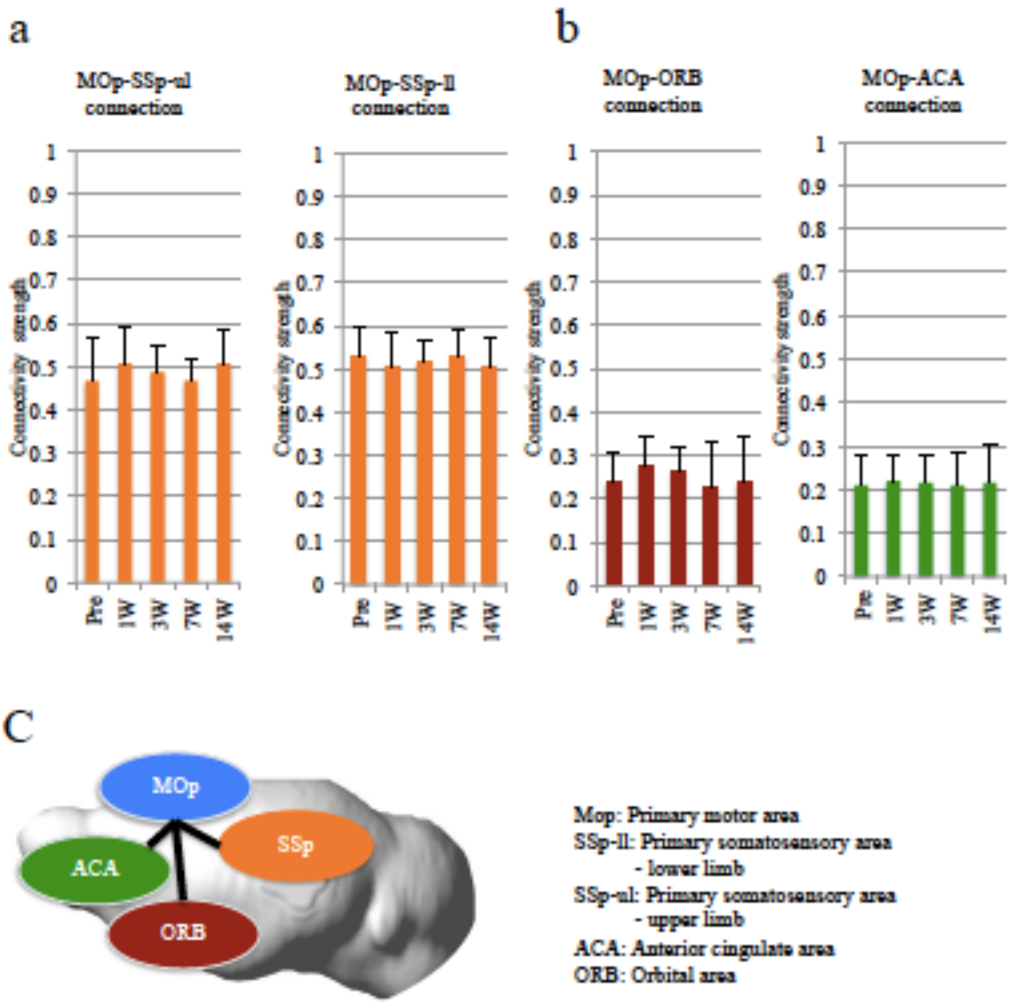

Supplement Figure 5

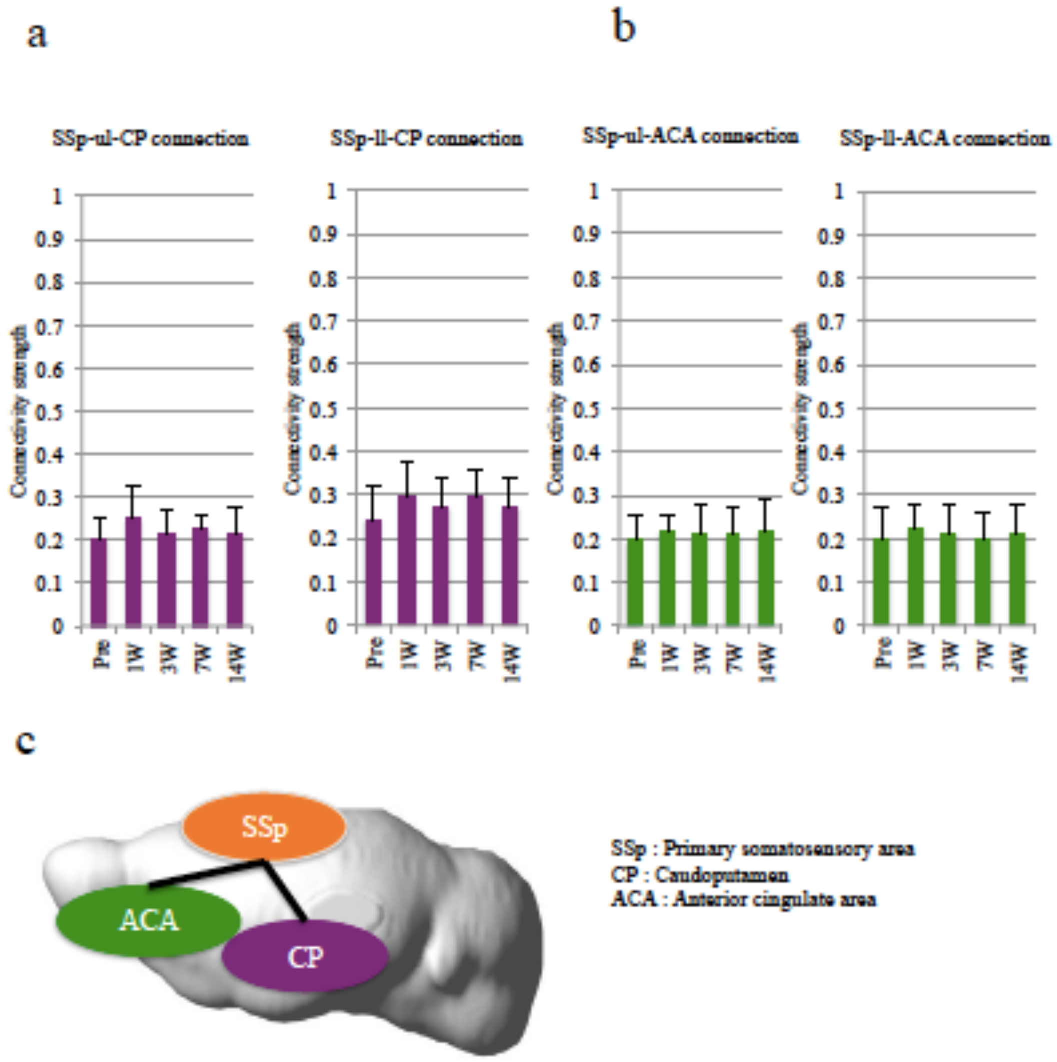

Supplement Figure 6

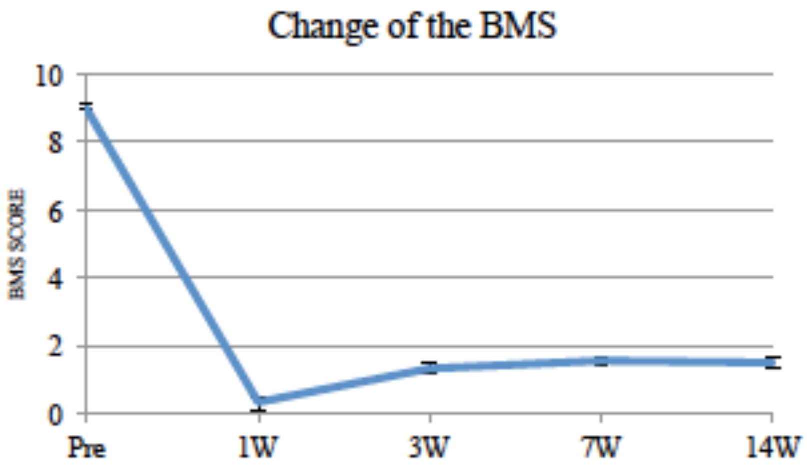

Supplement Figure legends

Fig. 1 Visualization of the neuronal functional connectivity in an awake mouse of the sham group .

***a**) The correlation matrix of awake mice with intact spinal cords expressed as the correlations among 576 areas.*

***b**) The brain networks were visualized on the basis of the correlation matrix of mice 14 weeks after the SCI.*

*Fig2**.** The change of the Characteristic path length and Clustering coefficient*

a) The change in the Characteristic path length

b) The change in the Clustering coefficient

*Fig. 3**.** Alterations in the connectivity in the entire brain of the sham group mice.*

***a**) The change in the number of connectivity in the entire brain.*

***b**) The change in the strength of the connectivity in the entire brain.*

*Fig. 4**.** The connectivity between the MOp and the SSp, ORB and ACA of the sham group mice .*

***a**) The change in the strength of the connectivity between the MOp and the SSp.*

***b**) The change in the strength of the connectivity between the MOP and the CP/ACA.*

***c**) A schematic of the connections among the MOp and the SSp, ORB and ACA.*

*Fig. 5**.** The connectivity between the SSp and the CP/ACA of the sham group mice.*

***a**) The change in the strength of the connectivity between the SSp and CP.*

***b**) The change in the strength of the connectivity between the SSp and ACA.*

***c**) A schematic of the connections between the SSp and CP/ACA.*

Fig.6 Change of the BMS in mouse SCI model.
